# Thoughts on the Action and Influence of the Nervous System, and on the Means of Strengthening and Improving Them

**Published:** 1840-09

**Authors:** Charles Caldwell


### THE
### WESTERN JOURNAL
### OF
### MEDICINE AND SURGERY.

---

SEPTEMBER, 1840.

---

**Art. I.**—*Thoughts on the action and influence of the Nervous System, and on the means of strengthening and improving them.* By Charles Caldwell, M.D.

The nervous system consists of the brain, the spinal marrow, and their appendages the nerves.

The nerves are divided into two great classes; nerves of sensation; and nerves of motion. The former of these classes contains numerous subdivisions; the latter but two, nerves of voluntary, and nerves of involuntary motion. Of these two subdivisions of the motory nerves, the latter is still farther divided into nerves of perceptible and nerves of imperceptible involuntary motion. Of perceptible involuntary motion we have manifestations in the actions of the lungs, heart, stomach, and intestines; of imperceptible, or organic motion, in the processes of secretion, nutrition, absorption, calorification, and growth.

The brain which constitutes the centre of the whole nervous system, but more obviously of the nerves of sensation and voluntary motion, consists of the cerebrum and cerebellum, or the larger and the smaller brain, separated from each other by the *tentorium.* By the falx, another process of the same membrane which forms the tentorium, the cerebrum is divided into two similar hemispheres, each of which is subdivided into thirty-six or seven minor portions called organs, constituting the seats or instruments of an equal number of different faculties of the mind. The organs and faculties of each hemisphere differ from one another; but the corresponding organs and faculties of the two hemispheres are alike. Each hemisphere therefore is composed of the same number of organs differing in function from one another, but identical in their several functions with the corresponding organs of the opposite hemisphere. Hence is the brain, in organization and action, a double viscus, in all respects, as it more palpably is with regard to the arrangement of the external senses.

The spinal marrow is made up of three portions or cords, an anterior, a posterior, and a lateral or central one. Of these the anterior cord is connected with the roots of the nerves of voluntary motion; the posterior with the roots, or rather with the terminations of some of the nerves of sensation; while the remaining cord or portion forms what Sir Charles Bell calls the nerves of respiration; but which Muller, Hall, and other physiologists denominate the *excito-motory*

nerves. Under this immediate head I shall only add, that though the sound condition of the whole nervous system is essential to the full health and efficiency of every portion of the body; it is the derangement of the nerves of imperceptible involuntary or organic motion, that produces the most serious and fatal complaints. They are the nerves, whose action is more literally indispensable to the *existence* of life; while the action of the other portions of the nervous system is immediately productive of little or nothing else than *modifications* of life—or rather modifications of vital action. But before proceeding farther in this discussion, a few remarks of a more general and abstract character are requisite.

There are three great sets or groupes of organs which constitute the basis of the being and character of man—which form I mean the most substantial and fundamental part of him, and contribute most essentially to make him what he is. These are the contents of the three great cavities of the body, and their appendages—the cranium, the thorax, and the abdomen. In plainer terms, they are the brain, spinal marrow and nerves, the lungs, heart and blood-vessels, and the chyle-making viscera consisting of the stomach and intestines, the liver, pancreas, and lacteal apparatus.

These three groups of organs are intimately connnected with and dependent on each other, in a twofold way; through sympathy or consent of parts; and through function. On the former ground, the condition of either group, whether morbid or healthy, produces a like condition in one or both of the others; and, on the latter, a derangement or failure of function in one, occasions in the others a corresponding derangement. This connexion and dependence are only stated as facts, no effort being made or intended at present to adduce their causes.

Although these sets of organs are each equally essential to human existence, and equally dependent on one another for soundness and efficiency, they hold different ranks, as respects their functions. The abdominal organs, being fitted only for digestion and chylification, are of the lowest order, and belong in some shape to the lowliest beings of the animal and vegetable kingdoms. The organs of the thorax, being framed for the maturation and circulation of the blood, are of a higher rank, and belong to a higher description of animals. But the cerebral, spinal, and nervous organs, ranking in an order still superior, belong to beings of superior classes, and, in their highest perfection, bestow on man his noblest attributes, and place him at the head of the animal creation. It is plain therefore that the improvement of the nervous system, to the utmost pitch of which it is susceptible, should constitute the leading object of all sorts of education and training. And on the attainment of that object depend the future standing, achievements, and happiness of our race, and the peace, prosperity, and glory of the world.

Is any one inclined to regard this statement as exaggerated at least, if not actually erroneous; and to ask me, whether it is not from the cultivation and improvement of the *mind* that results so beneficial and resplendent are to issue? Were this question put to me, my reply to it would be affirmative; but I would add, that the improvement of the nervous system, especially of the brain, and the improvement of the mind, are the same.

We have no ground to believe, much less are we authorized to assert, that the mind, as an independent and insulated being, is improved, or, in the slightest degree altered by education and training. Indeed the alteration, either for better or worse, of a spiritual substance, by any kind of action or influence on it that can be practised by us, is an event of which no conception can be formed. The thought is too subtle and etherial for imagination to grasp. Far less do we know what means to employ for the production of the effect. Nor is such an effect necessary for the attainment of the object aimed at in mental education. The improvement of the brain, the organ or instrument with which the mind works in all its operations, is sufficient for the accomplishment of the end held in view. And that that can be effected, by the suitable employment of suitable means, is an issue as certain, as that any other portion of the body can be improved.

The brain is but a mass of living organized matter, and like muscles, membranes, and all other similar masses, can be ameliorated with ease in temperament and tone, activity and power. And when thus advanced in the excellence of its qualities as a mental instrument, it follows of necessity that the mind must employ it with superior effect. This is as much of an absolute truism, in its bearing on the relation between cause and effect, as that a harp of higher finish and tone, when swept with the same skill and power, discourses more "exquisite music," than an instrument of an inferior order when out of tune.

As respects the process best fitted to improve the brain in activity and power, it is too plain to be mistaken, and too perfectly in accordance with reason and experience, to be made a subject of doubt, much less of controversy. It consists in giving to the organ a due amount of suitable exercise, and supplying it sufficiently with wholesome and well arterialized blood. And to do this is not only practicable, but exceedingly easy. That the end, however, may be fully and certainly attained, it is necessary that the brain be properly exercised in *all* its organs—its *affective* as well as its *intellectual.*

Partial exercise, so far as it *is* partial, must be comparatively fruitless. On this topic I shall only farther observe, that it is not, in mathematics, a more incontestible truth, that parallel lines can never meet, than it is in physiology, that exercise, proper alike in kind, quantity, and time of employment, increases the strength and activity of living organized matter of every description.

The questions then that first present themselves are, what is meant by the exercise of the brain? and in what way may such exercise be given to it? Nor is there any difficulty in answering them. The exercise of the brain is the heightened organic action of it; and that is produced by every form of salutary excitement. To exercise the mind is to exercise the brain, whatever may be the kind of mental operation. Is the mind actively engaged in vision, hearing, perception, observation, remembering, imagining, reasoning, thought, calculation, or in any sort of moral or religious feeling? The brain is simultaneously exercised in the organs corresponding to the kind of mental action pursued. Does the mental action consist in the indulgence of any of the passions or emotions?—in love or friendship, resentment or desire of revenge, in ambition, covetiveness, hope, wonder, or the contemplation and enjoyment of the sublime or the beautiful? In each and all of such cases does the brain conform in exercise to that of the mind. And its organs thus excited to action are invigorated and improved in aptitude for the performance of their functions.

That the brain may be the more certainly and competently strengthened and improved, I have said that it must be liberally supplied with arterial blood. And this end is more or less accomplished by the excitement it undergoes. "*Ubi irritatio, ibi affluxus*"—in whatever part of the body irritation or local excitement occurs, to that spot takes place a conflux of blood. And this is a maxim of as unquestionable truth, as is that which asserts, that things equal to one and the same thing, are equal to one other. Hence in cases of deep passion, such as anger and joy, (which are nothing but high cerebral excitement and action) the countenance is flushed and full, the eyes become fierce or sparkling and prominent, and at times the head turns painful and giddy, in consequence of the rush of blood to the parts. And in these instances the energy and power of action imparted to the brain, are strikingly manifested, by the unwonted force and rapidity of conception and thought, and the augmented fluency and eloquence of utterance, which mark the occasion.

Not only moreover does the blood flow to the brain in a larger volume; it is also more highly arterialized, than usual. Of this the reason is sufficiently obvious. The high and powerful action of the brain produces through sympathy a like condition in the lungs and heart; which latter organ, by its strengthened and accelerated pulsations, pours into the former an augmented stream of blood. Here again there is an increase of action. Respiration becoming more frequent and full, a larger amount of air is received into the lungs. The blood is hence more highly arterialized, and, passing in this condition to the left side of the heart, is thrown in greater force and quantity into the brain, the more thoroughly to vitalize, excite, and invigorate that viscus. Thus do the viscera of those two cavities, the skull and the thorax, operate on each other in a circle, or in a flux and reflux of influence, to the mutual increase of their power and action. Nor is this all.

The viscera of the third great cavity, the abdomen, which prepare the chyle, participate extensively in the general work.

Daily experience and observation testify, that, both through function and sympathy, the digestive organs are closely and powerfully connected with the heart, lungs, and brain. Without a sufficient amount of sound and well matured chyle, wholesome blood cannot be prepared in the quanity required for the uses of the system. And without such a stock of blood, neither vivification nor nourishment can be competently effected, and the brain, in common with other organs, must fail in its functions. Be the substance and nature of the vital principle, what they may, and from whatever source that principle may be derived, the arterial blood possesses it, and implants it in the solids of the body. Hence, other things being alike, the higher the pitch to which the vitalization of the blood is carried, the higher is the vitalization of the whole system; and, as far as consists with health, the greater the accumulation of arterial blood in any particular portion of the system, the higher is the vitality of that portion, and the more vigorous is its action. And this is as true of the brain and whole nervous system, as of other organs. That the brain therefore may possess the fullest amount of vitality, and the amplest power of action, of which it is susceptible, it must be supplied with as large an amount of well arterialized blood, as comports with its health. To express myself in plainer and more definite terms; what is true of every other organ, is equally so of the brain. To bestow on it the utmost power and activity of which it is susceptible, an actual orgasm, or state of vital erection must be produced in in it, by a thorough impregnation of it with arterial blood. The correctness of this position is easily proved. Passion, as already observed, consists in a state of the highest cerebral excitement that can co-exist with health; and it is the source of consummate mental energy and action. Let, therefore, a dog, a cat, a buck, or any other animal, be suddenly killed, during a paroxysm of rage, and its brain be examined, and the organ will be found almost as deeply injected with blood, as if it were in a state of actual inflammation.

To produce in the nervous system then, including the brain, the highest degree of power and activeness, of which it is susceptible, the work must be commenced in the chylopoetic organs. Those organs must be supplied with a suitable amount of wholesome and nutritious food. And care must be taken, that the amount be kept within the bounds of temperance. Thus will the organs, besides being invigorated themselves by salutary and natural exercise, produce, for the formation of blood, the necessary quantity of well-prepared chyle. For the conversion of this into wholesome blood, the lungs, in addition to a due degree of exercise and excitement by talking, declaiming, shouting, singing, and other forms of muscular action, must be liberally supplied with fresh and unvitiated atmospherical air. The same general muscular exercise that gives strength and activity to the lungs, produces a similar effect on the heart, and qualifies it for a vigorous circulation of the blood. And by such circulation the brain is injected with arterial blood sufficient in amount to invigorate it and prepare it for the extension of its influence, in return for what it has thus received from them, to the lungs, heart, and digestive organs, in common with all other parts of the body.

Such are the mutual influences and dependencies of the three leading groupes of organs of the body, by means of their functions. Their connexions by sympathy are equally stable, and their influences on one another, through that channel, more prompt and powerful in their immediate effects. Where one individual perishes from derangement of the functional influences of those organs, (that of hemorrhagy alone excepted,) thousands perhaps perish by the action of their sympathies. And the same is true in relation to recoveries and cures. The number of diseases removed by sympathy is immeasurably greater than the number successfully treated through the medium of function—but when both are brought into action, the influence is the greater, and the issue the more speedy, certain, and salutary.

But I have not yet stated fully and circumstantially what I mean by the phrase, "the action and influence of the nervous system." A few remarks to that effect therefore will now be in place.

This subject presents itself in several different divisions and bearings, and might be somewhat extensively considered under each of them, though my observations at present respecting it must be brief. It stands equally related to health and disease; and, in each of those conditions of the system, is connected alike with the operations and phenomena of the body and the mind. It is equally concerned moreover with the production, prevention, and cure of disease.

In health the range of nervous influence is co-extensive with that of the body itself. In every part of the system it is manifested and felt. Its most striking manifestation, as respects the body, is made by muscular motion, voluntary and involuntary. More or less however it is concerned in every form of vital action—I mean in the system of man, and in the systems of those animals that most nearly approach him in organization and character. In vegetables, and in many of the lower tribes of animals, a system of nerves exerts no influence, because it has no existence in them. In man however, and in other grades of animated nature where that system is developed, its influence takes part in digestion and chylification, absorption, nutrition, the arterialization of the blood, calorification, sleeping, waking, and dreaming, secretion, excretion, and growth. Were this statement contested, its proof would be ready; and, as part of it, the following may be received as satisfactory and conclusive. Destroy or paralyze the nerves belonging to any part of the body, and the processes just specified are impaired or extinguished in it.

In disease the nervous system is deeply involved in apoplexy, palsy, epilepsy, St. Vitus's dance, hysteria, tetanus, hydrophobia, colic, and every other form of convulsive and spasmodic affection. In these complaints it is chiefly the brain, spinal marrow and cerebro-spinal nerves that are concerned. They at least are most *obviously* and *strikingly* concerned. And in all febrile diseases, whether slight or severe, the ganglionic nerves are necessarily deranged. There is reason to believe, that in every complaint, some portion of the nervous system is *primarily* assailed, as constituting essentially the out-posts of the body. Nor is it less true that all curative articles make their first impression *immediately* on the nerves, especially the ganglionic nerves, which are instrumental in the production of *organic susceptibility;* and the impression passes to other parts of the system chiefly by sympathy.

In its connexion with the mind, the nervous system, especially the brain, both prevents and produces a variety of diseases. That organ, as has been heretofore observed, is the immediate instrument of the mental affections. And in protecting the system from certain complaints, and also in their production, some of those affections have great influence. Thus, during the prevalence of yellow fever, oriental plague, Asiatic cholera, and other epidemics, habitual timidity predisposes to those maladies, and a paroxysm of fear very often excites them. And calmness, firmness, and steady resolution are among their most efficient preventives. The operation of hope, by producing habitual cheerfulness, and a belief in the probability of escape, is also a very valuable safe-guard, unless it give rise to a want of precaution, leading to uncalled-for and perilous exposure. Hence, during the devastation of pestilential epidemics, those individuals (and for the honor of our race they are numerous) who calmly and fearlessly mingle with the sick, and minister with kindness and assiduity to their wants—such persons I say very generally escape the malady; while the fearful and the agitated, who shut themselves up in their own dwellings, shrink from the approach of a nurse or a physician who has visited in the house of sickness, turn pale at the sight of a herse or a coffin, and are startled by the sound of a funeral bell—these tremblers are far the most uniform subjects of attack. The reason is plain. Fear debilitates their systems in all respects, and very especially in their prophylactic powers. To such an extent is this true, that the heroic philanthropists, here alluded to, have been *often* supposed by the multitude, and *always* by the most credulous portion of it, to be in possession of some protective medicine or talisman, or to be under the immediate protection of Heaven. But, as respects either reputed source of safety, the supposition is not only groundless, but superstitious and silly. The prophylactic charm is altogether mental. It is a compound of calmness and prudence, temperance, resolution, cheerfulness, and active engagement. I say "active engagement;" for, on such occasions, *action* is infinitely preferable to *inaction*. The former strengthens the system in all its powers; while the latter enfeebles it. And those who are engaged and interested in business, have no leisure to listen to the alarming narratives of others, or to indulge their own apprehensions of danger. In proof of the correctness of these remarks, facts and anecdotes might be derived in abundance from the lives and histories of fearless philanthropists, who, in pestilential periods, have defied danger and death, in the cause of humanity, and escaped with impunity. That there may be something in the corporeal organization and temperament, which renders some individuals less susceptible of certain forms of disease than others, is altogether probable. But, in the cases referred to, I cannot doubt that escape is ascribable chiefly to the constitution of the mind.

Disappointed love, grief, the bitterness of violated friendship, jealousy, blighted ambition, wounded pride, the canker of remorse, nostalgia or frustrated longing of absentees to return to the delights of their native countries and their homes, and mental dejection, from whatever cause it may arise, are often productive of serious disease. So are violent paroxysms of the stronger and stormier passions, such as rage, the lust of vengeance, and even an excess of sudden and unlooked-for joy. And all these are but so many manifestations of the existing condition of the nervous system.

The complaints resulting from nervous action correspond in character to the action itself. Is the action sudden, violent, or acute? So are the diseases produced by it. Thus a fit of rage, an abrupt and overwhelming eruption of joy or sorrow, terror or despair, give rise to apoplexy, palsy, hysteria, epilepsy, and other forms of convulsion, and also to hemorragy from the rupture of large blood vessels, or even of the heart itself, and at times to an immediate extinction of life. In the latter case the shock received operates on the brain and nerves like the crush of a falling and ponderous body, or like a blow on the head from a massy weapon and a powerful arm.

In like correspondence with their nature and character, disappointed love and ambition, moderate but protracted grief, remorse, nostalgia, and other chronic and cerebral affections are productive only of chronic complaints. Of these I may specify, as among the leading maladies thus engendered, madness, dyspepsia, chronic hepatitis, jaundice, general tabes and pulmonary consumption. Other insidious and wasting affections might be added. But, as concerns the instrumentality of nervous influence in the *production* of disease, the foregoing examples are believed to be sufficient for illustration and proof.

The *therapeutic* influence of nervous action is yet to be considered. This is denominated "moral medicine," and is a subject not only extensive in its limits, rich in matter, and abundantly curious, but highly important in the treatment of disease.

As the phraseology itself implies, moral medicine consists in the production of moral or mental impressions immediately on the brain, through the medium of the senses. To render the practice successful, these impressions must be such as are best calculated to produce in the brain a healthful condition. That organ, possessing as it does, a predominant influence over the other parts of the body, does much, when sound itself, to communicate soundness to the entire system. Hence the great importance of keeping it, in all cases, as far as possible, in a state of health. The degree of action moreover maintained in it must be judiciously adapted to the character of the complaint under treatment. That it may prove a salutary remedy, it must be employed with skill, as respects both quantity, manner, and time. If the disease be inflammatory, or one of high excitement without inflammation, the brain should be kept tranquil, more especially if the affection be in any degree cephalic. But in complaints of languid action, the tone and excitement of the brain should be heightened.

This practice is more extensively and perhaps more skilfully pursued in France, than in any other country. Nor is it improbable that, from the superior sensitiveness of their systems, the native French population are more susceptible of benefit from moral medicine, than any other people. And next to them perhaps, in this respect, come the Irish and the natives of the United States, who are certainly a more susceptive and impressible people, than either the British, Hollanders, Belgians, or Germans. Hence the propriety and usefulness of cultivating and employing this form of practice among ourselves.

The forms of disease which moral medicine, when skilfully administered, always soothes and relieves, and often removes, are numerous, and many of them of a grievous and dangerous character. Only a few of them, however, can be specified at present.

Among these, *insanity* holds the most conspicuous place ; because it assaults and deranges or entirely destroys what constitutes, not only the highest and brightest attribute, but the very essence of human nature. For the understanding and rational treatment of this malady, an acquaintance with phrenology is indispensable—as literally and positively so, as an acquaintance with the anatomy and physiology of the thoracic viscera is to the rational treatment of complaints of the chest.

The ground of this assertion is plain and substantial. In a vast majority of cases, if not in every one, insanity, at its commencement, is a *local affection.* It is, I mean, a derangement of but *one* or *a few* of the cerebral organs and their functions. For it is not now to be made known that madness is as entirely and essentially a disease of organized matter (the mind as a separate substance or entity being perfectly uninjured) as is pneumonitis or rheumatism. And in perhaps every case, I say, that disease begins in one or two organs, and spreads, with more or less rapidity, until a larger portion of the brain becomes involved.

Nor can any one but a physician acquainted with prenology discover, at this early period, when alone the evil is *certainly* tractable, the exact seat of the complaint, and ascertain to what extent the brain is deranged. No other physician therefore is so well prepared to encounter the malady with the proper form of treatment; especially of moral treatment. But I must dwell on this point no longer. My object is not to write an essay on the cure of madness; but to show that it is a complaint, to whose cure moral medicine is peculiarly adapted; and that, other things being alike, the phrenologist is best qualified to administer that medicine. And if the deranged organs be too highly excited, the proper moral administration consists in reducing that excitement, by bringing them into a state of rest, and transferring their action to other organs, on the principle of revulsion. To illustrate my meaning by a familiar example.

Has the patient induced his malady by intense application to a favorite study? Let him abandon that study, and engage in some other less arduous and severe, and which will give suitable exercise to other cerebral organs than those which he has overworked and injured; or let him relinquish for a time all real study, and enjoy relaxation and pleasurable exercise both mental and corporeal, in travelling, hunting, gardening, or some other amusing pursuit, and in social intercourse with his family and friends. By such treatment, if adopted in time, and duly persevered in, may insanity be averted with as much certainty and ease, as any other malady can be thrown off, before it has fairly taken root in the system. And by such means it frequently has been averted.

*Nostalgia* is another complaint within the control of moral medicine. And its treatment is simple. Let the sick return to their country and home, and then is recovery certain. This is but giving rest to an over-excited cerebral organ, and is analogous to the treatment and cure of a sprained ancle-joint, or a fractured limb, by quietude, ease, and soothing applications.

The disease produced by *disappointment in love* is in like manner within the reach of moral medicine. Nor is there the slightest difficulty in its successful treatment, provided the curative means can be applied. And those means may be stated in a few words. They consist in a return of affection by the beloved object, and the rites of matrimony. Except in cases where some confirmed and fatal *secondary* affection is produced by delay, these remedies never fail.

In the cure of *dyspepsia* moral medicine, skillfully employed, is peculiarly efficacious. Indeed if the complaint, as is very often the case, be induced by mental causes, that form of medicine is indispensable, and sufficient of itself, provided its application be opportunely made.

In the treatment of this disease, the removal of care, grief, and all other forms of mental perplexity, and the cheerfulness and serenity thus produced, are among the most useful and important of remedies. Add to them out-of-door exercise, temperance, and the influence of a salubrious atmosphere, and the complaint can hardly fail to give way. Hence the benefit derived by dyspeptics from travelling, a sojourn at watering places, and sea-voyages, where the operation of all these remediate agents may be united.

To speak on this subject in general terms. In the treatment of every form of disease resulting in any measure from cerebral derangement, the employment of moral medicine is essential to success. The reason is obvious. To do away an effect, you must first do away the cause. If therefore any sort of cerebro-mental influence has produced the complaint, such influence must be removed, else the complaint will remain. And by moral medicine only can the removal be effected.

As heretofore intimated, all persons are not so constituted, as to derive the same degree of benefit from moral medicine. Different individuals are endowed with different shares of sensitiveness and impressibility. And, other things being equal, the effect of moral treatment will be in direct proportion to the impressibility possessed. Hence, for receiving benefit from such treatment, women are more suitable subjects than men; because their sensibility is greater. And, for a similar reason, the youthful of the same sex are more suitable than the older.

In the cure of all chronic, debilitating, and depressing complaints, the agency of hope, resolution, and cheerfulness, faith in remedies, and confidence in physicians, is efficacious and powerful. And all these belong to moral medicine. So does the influence of bravery and ambition in the soldier and the sailor. And that has removed, at times, some of the most grievous and obstinate of maladies. The following instances are in proof of this; and many other similar ones might be added.

During the last war between the United States and Great Britain, a memorable battle was fought by the fleets of the two nations, on Lake Erie, in the year 1814 or '15. For about forty-eight hours (I think) before the action the two squadrons were in sight of one another, manœuvring each to gain the windward quarter. At the time when the British squadron first made its appearance, and the occurrence of an engagement was no longer doubtful, a full third part, if not more, of the American officers and sailors were confined to their berths, by severe illness, or its debilitating effects. Their complaint was the *lake-fever*. But such was the effect of their desire to come to action with the enemy, and of their ambition and determination to vanquish him, that, at the commencement of the conflict, nearly every man was at his post, prepared to do his duty. The result was a decisive and glorious victory.

Near the close of our revolutionary war, a similar event occurred, in the great naval action, fought not far distant from the American coast, between the French and British fleets, the latter commanded by admiral Rodney, and the former by the Count de Grasse. When the two fleets first descried each other, and an approaching conflict was certain, a very large portion of the British crews were confined to their hammocks by scurvy. From the manœuvring which followed, however, between the parties, each striving to gain the wind of the other, two days or more elapsed, before they came to action. During this interval, so salutary on the British sailors was the influence of their courage, ambition for battle, and confidence of victory, that, when the engagement commenced scarcely a man was absent from his gun. The issue is known. The French fleet was almost annihilated.

Again. In the disastrous retreat of the French army from Moscow, thousands on thousands of the soldiers are known to have perished, exhausted by fatigue and hunger, and stiffened by cold. And when, from the insupportable and agonizing agency of those three destroyers, whole regiments were ready to sink to the earth, and there expire, the approach and assault of the Russians or Cossacks on their rear or flank, never failed to revive and arouse them to a desperate and vigorous resistance. And no sooner was the enemy repulsed, than they all returned to their enfeebled condition; and hundreds of them, not from recent wounds, but from exhaustion and torpidity, dropt at the feet of their comrades, to rise no more. So powerful on their wasted and enfeebled systems, was the influence of the pride of soldiership, invincible bravery, and an ambition to conquer.

Once more. The following fact, transmitted to us on respectable authority, is well worthy of the attention of all physicians, but more especially of those of the army and navy.

"In the year 1625, the city of Breda suffered, from a long seige, all the miseries that fatigue, bad provisions, and distress of mind could bring on the inhabitants. Among other misfortunes, the *scurvy* made its appearance, and carried off great numbers. This, added to the other calamities, induced the garrison to incline towards a surrender of the place; when the Prince of Orange, anxious to prevent its loss, and unable to relieve the garrison, contrived, however, to introduce letters addressed to the men, promising them the most speedy assistance. These were accompanied with medicines against the scurvy, said to be of great price, but of still greater efficacy; many more were to be sent there. Three small vials were given to each physician. It was publicly given out, that three or four drops were sufficient to impart a healing virtue to a gallon of water. We now displayed," (says the narrator, who was himself a physician belonging to the garrison, and engaged with his colleagues in the moral practice—for it was nothing more)—" we now displayed our wonder-working balsams. Not even were the commanders let into the cheat practised on the soldiers. They flocked in crowds about us, every one who had the scurvy soliciting that some part might be reserved for his use. *Cheerfulness* again appears in every countenance, and an universal *faith* prevails in the sovereign virtues of the remedies. The effect of this *delusion* was truly astonishing; for many were quickly and perfectly recovered. Such as had not moved their limbs for a month before, were now seen walking the streets with their limbs sound, straight, and whole. * * * Many, who had declared that they had been rendered worse by all former remedies, recovered in a few days, to their inexpressible joy, and the no less general surprize, by taking what we affirmed to be their gracious Prince's cure." From this occurrence, the drug, whatever it might have been, was afterwards known by the name, sometimes of the "Prince's," and sometimes of the " Commander's Balsam. "

It is by the like operation of *hope* and *cheerfulness*, confidence in their own skill and faith in their remedies, that quacks perform many remarkable cures, in cases where educated physicians, employing no moral means, had practised unsuccessfully. Nor is this all.

Homœopathy is virtually a scheme of moral medicine. It is not to be fancied, even by the most enthusiastic credulity, that the material remedies administered by Hahnemann and his followers, in doses so infinitely minute, can have the slightest influence in the cure of diseases. Yet, that cures, at times very remarkable, are produced by their practice, is not to be denied· To what then is their success attributable? The answer is easy. It is the product of several causes—a temperate and well directed diet and regimen, which they strictly enjoin; and of cheerfulness, hope, and a firm belief in recovery, which they sustain in their patients, by their confident and unqualified assurances of success. Thus do they produce and maintain in the brain a sound and salutary condition; while that organ extends a healing influence throughout the system.

Other things being alike, moral remedies act most powerfully and successfully on persons of an active temperament, whose organs of Hope and Wonder, Benevolence, Ideality, and Firmness are largely developed. Hence; as respects this form of practice, the beneficial effects of an acquaintance with phrenology. It enables the physician to detect in his patients their greater or less fitness for moral treatment.

